# Comparative clinical characteristics among different age group of adult COVID‐19 patients: A multicenter study

**DOI:** 10.1002/iid3.550

**Published:** 2021-10-28

**Authors:** Wei Cheng †, Yating Peng †, Aiyuan Zhou, Ling Lin, Xin Liao, Dingding Deng, Peng Huang, Wenlong Liu, Mingyan Jiang, Xudong Xiang, Qingcui Shuang, Shan Cai, Ping Chen, Xucai Liao

**Affiliations:** ^1^ Department of Respiratory and Critical Care Medicine, Second Xiangya Hospital Central South University Changsha Hunan China; ^2^ Department of Pulmonary and Critical Care Medicine, Xiangya Hospital Central South University Changsha Hunan China; ^3^ Department of Respiratory Medicine Affiliated Shaoyang Central Hospital of University of South China Shaoyang Hunan China; ^4^ Department of Respiratory Medicine The First Attached Hospital of Shaoyang University Shaoyang Hunan China; ^5^ Department of Respiratory Medicine Zhuzhou Central Hospital Zhuzhou Hunan China; ^6^ Department of Respiratory Medicine, Yueyang Second People's Hospital Designated Hospital of Junshan District Yueyang Hunan China; ^7^ Department of Respiratory and Critical Medicine Xiangtan Central Hospital Xiangtan Hunan China; ^8^ Department of Emergency Medicine, Second Xiangya Hospital Central South University Changsha Hunan China

**Keywords:** age, COVID‐19, critical, severe

## Abstract

**Background:**

Coronavirus disease (COVID‐19) is a global infectious disease with a large burden of illness and high health care costs. This study aimed to compare clinical features among adult COVID‐19 patients in different age groups.

**Methods:**

Laboratory‐confirmed adult COVID‐19 infection cases between December 31, 2019 to March 8, 2020 obtained from Neighboring Cities. Patients were divided into five age groups. Clinical characteristics were compared among different age groups.

**Results:**

Of 299 cases, median age was 44 and 158 (53%) were male. A total of 53.3% of 30–40 years, 50% of 40–50 years, 36.6% of <30 years and 36.2% of 50–60 years were primary case, none of the elderly were primary case. Among all the observed symptoms, only symptom of dyspnea was significantly different between the elderly group and other groups (*p* < .001). Proportion of severe or critical type was 2.4%, 5.3%, 9.5%, 14.5%, and 35% in patients with age <30, 30–40, 40–50, 50–65, ≥65, respectively. A total of 285 patients (95.3%) were cured and discharged, 12 patients (4.0%) were still on medical treatment in hospital. There were 2 (0.7%) deaths which occurred among persons ≥65 years. Patients with a history of chronic heart disease had a more than a 56 times higher risk for severe or critical type of COVID‐19 than those without a history of chronic heart disease (odds ratio [OR]: 56.038, 95% confidence interval [CI]: 2.764–1136.053, *p* = .009). Old age (OR: 1.055, 95% CI: 1.016–1.095, *p* = .006), high heart rate in admission (OR: 1.085, 95% CI: 1.03–1.144, *p* = .002), high respiratory rate in admission (OR: 1.635, 95% CI: 1.093–2.431, *p* = .017) were independently associated with severe or critical type in COVID‐19.

**Conclusions:**

Proportion of severe or critical type increased with old age groups. Adults with old age and high heart rate, respiratory rate in admission and history of chronic heart disease were associated with severe or critical type in COVID‐19.

## Graphical abstract

This study aimed to compare clinical features among adult COVID‐19 patients in different age groups. Among all the observed symptoms, only symptom of dyspnea was significantly different between the elderly group and other groups. Old age, high heart rate on admission, high respiratory rate on admission, and history of chronic heart disease were independently associated with severe or critical.

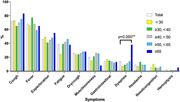

## 1 INTRODUCTION

The current outbreak of coronavirus disease (COVID‐19) pandemic that was first reported from Wuhan, China, on December 31, 2019 has developed into a world‐wide public health emergency. On February 26th, 2020, the World Health Organization (WHO) announced that the number of new cases of COVID‐19 reported outside China had exceeded the number of new cases in China. And then they declared this viral disease a pandemic on March 11, 2020. Globally, as of 17th July 2021, there have been 188,655,968 confirmed cases of COVID‐19, including 4,067,517 deaths, reported to WHO.^1^ In China, epidemiological evidence suggested that most of these patients had travelled to Wuhan city (Hubei province) before the onset of illness.^2^ Around China's spring festival in early February, rapidly increasing new cases were identified in Hubei and neighboring provinces and cities. Until 17 July 2021, cumulative reported confirmed cases was 119,614 in China, and cumulative deaths were 5612, while cumulative reported confirmed cases was 1068 in Hunan province.^3^

Severity assessment is the key to determining patient management, appropriate treatment, and resource allocation in epidemic events. For example, in China's experience, the Fangcang Hospital in Wuhan city is organized to treat large numbers of patients with mild and moderate COVID‐19, whereas other facilities with negative‐pressure isolation wards and intensive care units (ICU) serve patients with severe and critical COVID‐19. Incidence and mortality of community‐acquired pneumonia (CAP) is much greater in the elderly (>65 years) than in younger populations.^4^ Old age itself is an independent risk or prognostic factor for pneumonia.^5^ More frequent comorbid illness, less specific presenting clinical signs, and lowered effectiveness of therapy in older subjects also add risk.^4^, ^6^

Patients of different ages may have distinct physiological characteristics, susceptibility, clinical presentations, and response to medical treatment.^7^, ^8^, ^9^ Therefore, age‐ specific risk factors for disease severity may be useful for clinical management. To the best of our knowledge, there has been no comprehensive study regarding exploration of the associated factor of severe or critical severe type on COVID‐19 cases with different age group. We report here findings describing the influence of age on clinical and laboratory features from a cross‐sectional study of patients treated for COVID‐19 in six University or municipal hospitals in Hubei and Hunan provinces.

## 2 MATERIALS AND METHODS

### 2.1 Study participants and procedures

A hospital‐based COVID‐19 disease surveillance program was established and maintained from December 2019 to March 2020. This included 6 participating hospitals (Affiliated Shaoyang Central Hospital of University of South China, The first Attached Hospital of Shaoyang University, Zhuzhou Central Hospital, Designated Hospital of Junshan District, Xiangtan Central Hospital) from Hunan Province and Puyang District People's Hospital from Hubei Province. We included patients ≥18 years who were positive for coronavirus nucleic acid detection in throat swab at admission. None of the patients were positive for Influenza A or B virus nucleic acid detection in throat swab.

This is a cross‐sectional study based on review of medical records. Clinical variables were collected on standardized case report forms at each hospital. The research protocol was approved by the ethics committee of the Second Xiangya Hospital (No. 2020‐010) and was approved by all other participating hospitals. We have been obtained oral consent from patients that their medical records could be potentially used for studies in the future. And then the informed consent was waived in this study approved by the ethics committee of the Second Xiangya Hospital.

### 2.2 Definitions

Patient variables were evaluated for all cases: Demographic characteristics (e.g., age, sex, body mass index); chronic disease history involving the central nervous system, cardiovascular system, lungs, liver, kidneys, endocrine system, autoimmune conditions; initial vital signs (e.g., temperature and respiratory rate), laboratory test results (white blood cell [WBC], lymphocyte [Lym], neutrophil [Neu], platelets [Pt], hemoglobin [Hb], Prothrombin time [PT time], activated partial thromboplastin time [APTT], D‐Dimer [DD], albumin, alanine aminotransferase [ALT], aspartate aminotransferase [AST], creatine kinase [CK], creatine kinase isoenzyme [CK‐MB], total bilirubin [TBIL], K^+^, Na^+^, urea nitrogen [BUN], creatinine [Cre], blood cell sedimentation rate [ESR], Lactate dehydrogenase [LDH], Myoglobin [Mb], Random blood glucose [Glu], C‐reactive protein [CRP], Procalcitonin [PCT], chest radiography); and medications. The data on initial vital signs, laboratory examinations and chest imaging results were derived from the intial results of the patients in the hospital.

For epidemiological history, patients with Wuhan travel history were defined as primary cases; patients without Wuhan travel history, but with contact with primary cases or confirmed cases, were defined as secondary cases, the remainder without definite epidemiological linkage were classified as “unknown”.

COVID‐19 severity was classified into four categories. Mild: mild clinical symptoms (cough, fatigue, dyspnea) without imaging findings of pneumonia. Moderate: fever (admission temperature ≥39°C and respiratory symptoms with imaging findings of pneumonia. Severe: having any of the following criteria: (1) Respiratory distress with respiratory frequency ≥30 breaths/min; (2) Resting oxygen saturation (SpO_2_) ≤93% on room air; (3) PaO_2_/FiO_2_ ≤300 mmHg (1 mmHg = 0.133 kPa). Critical: having any of the flowing criteria: (1) a respiratory failure in need of mechanical ventilation; (2) shock; (3) with other significant organ dysfunction.^10^

Outcome was classified as cured and discharged, undergoing treatment in hospital, and deceased in‐hospital. Patients were discharged from hospital upon meeting the following criteria: (1) body temperature returned to normal for more than 3 days; (2) Respiratory symptoms improved significantly; (3) Improvement in pulmonary radiographic findings; (4) Two negative throat swabs for coronavirus nucleic acid taken 24 h apart.^10^

### 2.3 Statistical analysis

Categorical variables were expressed as counts and percentages. Depending on whether it is normally distributed, continuous variables are expressed as mean ± *SD* or median, 25–75th interquartile range (IQR). Differences in frequencies were compared using chi‐square test or Fisher's exact test. In the comparison of continuous numerical variables in independent groups, the Mann–Whitney *U* test was used in the case of two groups, whereas nonparametric tests with multiple independent samples corrected by Bonferroni were used three or more groups. Factors with significant (*p* < .05) unadjusted associations with disease severity and suspected important variables were included in subsequent multivariable logistic regression analysis (Forward method) to determine risk factors associated with disease severity, yielding adjusted odds ratios (ORs) and 95% confidence intervals (CIs). Goodness of‐fit was tested using the Hosmer‐Lemeshow test [reference]. All statistical tests were two‐sided, and *p* < .05 was considered to be statistically significant. All analyses were performed using IBM SPSS Statistics version 18.0 for Windows (IBM Corp.).

## 3 RESULTS

### 3.1 Demographic and clinical characteristics

**Figure 1:**
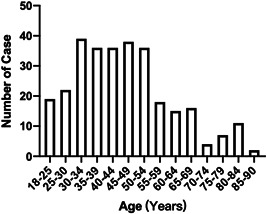
The distribution of patients in different age group

Of 299 patients, ages ranged from 18 to 88 years with a median of 44 years (IQR: 20), and half of participants were male (Table 1). Half of patients were in the 30–50 years age groups (Figure 1).

**Table 1:** Baseline clinical characteristics of COVID‐19 according to age groups

	Total	<30	≥30, <40	≥40, <50	≥50, <65	≥65	
	*n* = 299	*n* = 41	*n* = 75	*n* = 74	*n* = 69	*n* = 40	*p* value
Age (year)	44 (34, 54)	25 (22, 28.5)	34 (32, 37)	45 (42, 47)	54 (52, 59)	74.5 (67, 80)	.000**
Sex (% male)	158 (53%)	27 (66%)	42 (56%)	43 (58%)	30 (43%)	16 (40%)	.061
Weight (kg)	65 (56, 74)	68 (53, 77.5)	65 (54, 75)	66 (58, 75)	64 (60, 70)	59 (50, 61)	.043*
Height (cm)	164 (158, 170)	170 (160, 175)	166 (160, 172)	168 (158, 170)	160 (156.5, 166.5)	158 (156, 164.5)	.000**
BMI (kg/m^2^)	23.38 (21.63, 26.08)	22.34 (20.05, 26.8)	23.13 (20.96, 25.16)	23.63 (22.38, 26.22)	24.06 (22.86, 26.37)	22.21 (20.36, 24.52)	.063
Total number of chronic disease	0 (0, 1)	0 (0, 0)	0 (0, 0)	0 (0, 1)	0 (0, 1)	1 (1, 2)	.000**
Temp (°C)	36.75 (36.5, 37.2)	37 (36.7, 37.3)	36.8 (36.5, 37.2)	36.7 (36.5, 37.23)	36.6 (36.4, 37.1)	36.6 (36.43, 37)	.026*
HR (min^−1^)	86 (78, 96)	87.5 (83.5, 100)	82 (76, 95.25)	85 (77.5, 92)	88.5 (82, 98)	90 (78.25, 99)	.036*
RR (min^−1^)	20 (20, 20)	20 (18, 20)	20 (19.75, 20)	20 (19.75, 20)	20 (20, 20)	20 (20, 21)	.025*
Chronic disease	105 (35%)	2 (5%)	12 (16%)	25 (34%)	36 (52%)	30 (75%)	.000**
DM	35 (12%)	1 (2%)	4 (5%)	7 (9%)	10 (14%)	13 (33%)	.000**
hypertension	53 (18%)	0 (0%)	2 (3%)	12 (16%)	16 (23%)	23 (58%)	.000**
Chronic heart disease	14 (5%)	0 (0%)	1 (1%)	2 (3%)	6 (9%)	5 (13%)	.013*
Chronic pulmonary disease	17 (6%)	1 (2%)	2 (3%)	1 (1%)	9 (13%)	4 (10%)	.011*
Cancer	5 (2%)	0 (0%)	0 (0%)	2 (3%)	2(3%)	1(3%)	.549
Chronic nervous system disease	8 (3%)	0 (0%)	2 (3%)	0 (0%)	4(6%)	2(5%)	.147
Chronic liver disease	8 (3%)	0 (0%)	3 (4%)	4 (5%)	1 (1%)	0 (0%)	.265
Chronic kidney disease	1 (0%)	0 (0%)	0 (0%)	0 (0%)	0 (0%)	1 (3%)	.134
Endocrine and immune disease	5 (2%)	0 (0%)	1 (1%)	2 (3%)	1 (1%)	1 (3%)	.878
Bilateral involvement on chest radiographs	236 (79%)	25 (61%)	55 (73%)	61 (82%)	59 (86%)	36 (90%)	.005**
Epidemiological history							
Primary case	117 (39.13%)	15 (36.59%)	40 (53.33%)	37 (50%)	25 (36.23%)	0 (0%)	.000**
Secondary case	111 (37.12%)	18 (43.9%)	20 (26.67%)	22 (29.73%)	29 (42.03%)	22 (55%)	
Unknown case	71 (23.75%)	8 (19.51%)	15 (20%)	15 (20.27%)	15 (21.74%)	18 (45%)	

*Note*: *p < .05, **p < .01.

Abbreviations: DM, diabetes mellitus; HR, heart rate; RR: respiratory rate; Temp, temperature.

One‐third of participants had one or more chronic disease. The total number of chronic diseases differed significantly (*p* < .001) between age groups, with elderly have more chronic disease. Older age groups had more patients with at least one or more chronic disease (5%, 16%, 34%, 52%, and 75% in <30, 30–40, 40–50, 50–65, and ≥65 age groups, respectively; *p* < .001). Older age groups had higher prevalence of diabetes mellitus (DM) history (*p* < .001) and hypertension history (*p* < .001), chronic heart disease history (*p* = .013), and chronic pulmonary disease history (*p* = .011). Prevalence of bilateral radio graphic pneumonia findings was high in all five age groups (overall, 79%) and was increased significantly with older age (61%, 73%, 82%, 86%, and 90% in <30, 30–40, 40–50, 50–65, and ≥65 age groups, respectively; *p* = .005). 71 (23.75%) patients could not provide a definite contact or exposure history with confirmed or suspected COVID‐19 infection. A total of 53.33% of 30–40 years and 50% of 40–50 years were primary cases, and none of the elderly were primary cases. Percentage of secondary cases were 55% among elderly, 42.03% in 50–65 years and 43.9% in <30 years. Temperature, heart rate, respiratory rate on admission were slightly increased in older age groups (Table 1).

### 3.2 Symptoms

**Figure 2:**
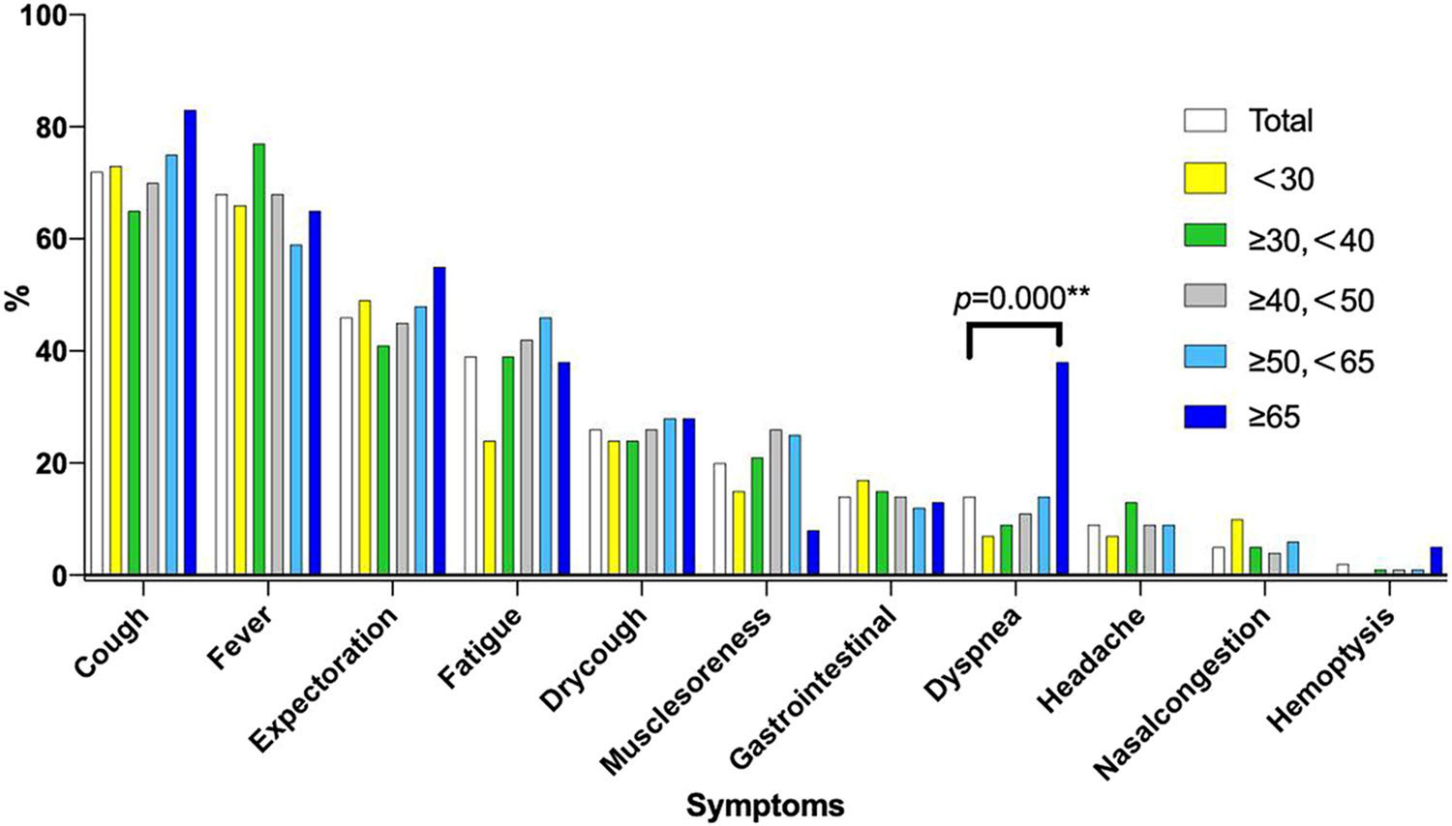
Symptoms of COVID‐19 according to age groups

The most common symptoms were cough in 216 (72%) patients, fever in 202 (68%), expectoration in 139 (46%), fatigue in 117 (39%), dry cough in 77 (26%) and were of comparable prevalence in all age groups. Only dyspnea was significantly different between elderly group and other groups (*p* = .001) (Figure 2).

### 3.3 Comparison of laboratory testing profile

As shown in Table 2, age ≥65 had significant lower lymphocyte percentage compared to age <30 and age 30–40 (19.25 vs. 32.75, *p* = .003; 19.25 vs. 29.1, *p* = .015). Age <30 had significantly higher levels of lymphocyte counts compared to age 30–40, 40–50, 50–65, ≥65 (1.6 vs. 1.2, *p* = .009; 1.6 vs. 1.15, *p* = .002; 1.6 vs. 1.29, *p* = .021; 1.6 vs. 1.06, *p* < .001). The level of ESR and PCT of age ≥65 were the highest among all groups; these values were higher than level of ESR and PCT of age <30, 30–40, 40–50, and 50–65 with statistically significant differences (*p* < .001). Age ≥65 had significantly higher levels of CRP compared to age 30–40 (13.9 vs. 5, *p* = .002). The decrease in lymphocyte count and the increase in ESR and PCT may be attributed to the dysregulation of the immune status and the intensified response to proinflammatory cytokines.

**Table 2:** Laboratory testing profile of COVID‐19 according to age groups

	Total	<30	≥30, <40	≥40, <50	≥50, <65	≥65	
	*n* = 299	*n* = 41	*n* = 75	*n* = 74	*n* = 69	*n* = 40	*p* value
WBC (×10^9^)	4.97 (3.86, 6.49)	5.34 (4.18, 7.82)	4.7 (3.64, 5.8)	4.79 (3.62, 5.84)	4.95 (3.97, 6)	5.59 (3.87, 7.73)	.151
N (×10^9^)	3.09 (2.32, 4.54)	2.95 (2.16, 4.23)	2.97 (2.31, 3.93)	2.98 (2.29, 3.96)	3.13 (2.34, 4.6)	3.86 (2.4, 6.17)	.242
*N*%	63.9 (56, 72.3)	57.8 (51.6, 66.7)	61.8 (56, 73.8)	65.1 (56.2, 71.9)	64.5 (55.5, 72.38)	69.25 (61.15, 82.53)	.006**
L (×10^9^)	1.22 (0.84, 1.63)	1.6 (1.23, 2.31)	1.2 (0.8, 1.62)	1.15 (0.85, 1.52)	1.29 (0.87, 1.64)	1.06 (0.63, 1.49)	.000**
L%	27 (19.3, 35)	32.75 (23.58, 42.93)	29.1 (21.8, 35.4)	26.1 (19.35, 35.95)	27.45 (19.85, 36.35)	19.25 (11.55, 27.6)	.003**
Hb (g/L)	134 (123, 147.5)	146 (132.25, 158)	140 (129, 150)	141 (129, 152)	126 (119, 138)	122.5 (118, 131)	.000**
Pt (×10^9^)	206 (161, 253)	245 (195, 273.5)	214 (172, 248)	216.5 (159.25, 259.25)	184 (146.5, 239)	175.5 (144.75, 228.75)	.01**
PT time (s)	12.3 (11.1, 12.99)	12.6 (10.83, 13.3)	12.3 (10.9, 12.8)	12.34 (11, 13.08)	12.03 (11.07, 12.9)	12.65 (11.73, 13.28)	.558
APTT (s)	32.7 (29.1, 37.2)	30.9 (27, 33.69)	34.12 (30.2, 38.14)	32.3 (29.4, 37.03)	33.9 (27.8, 37.1)	31.64 (28.7, 39.66)	.189
DD (mg/L)	0.32 (0.2, 0.49)	0.29 (0.13, 0.4)	0.28 (0.18, 0.4)	0.31 (0.2, 0.44)	0.35 (0.2, 0.5)	0.66 (0.4, 1.46)	.000**
albumin (g/L)	42.25 (38.57, 45.7)	45.36 (42.15, 49)	44 (41.22, 46.7)	42.6 (38.98, 45.72)	40.9 (36.6, 43.7)	36.92 (32.35, 40.4)	.000**
ALT (U/L)	21.6 (14.95, 35)	19.25 (13.08, 28.9)	22 (15, 38.7)	27 (16.98, 43.1)	22.4 (15, 34.3)	17.85 (12.58, 24.85)	.024*
AST (U/L)	23 (18.1, 30.8)	21.85 (18.25, 26)	22 (17, 29)	23 (19.68, 32.73)	24 (18, 30.9)	25.3 (19.33, 39.8)	.122
CK (U/L)	67.1 (46.08, 103)	72 (51.1, 100)	64 (48.4, 103)	63.5 (43.5, 109.5)	67 (45.5, 96.31)	88.5 (47.48, 145.23)	.419
CK‐MB (U/L)	8 (2.5, 11.93)	6 (2.5, 9)	9 (2.5, 11.93)	8 (2.5, 10.63)	8 (2.5, 11.8)	11 (4.85, 17)	.088
TBIL	11.55 (8.18, 18.49)	11.95 (7.4, 19.78)	11.9 (8.02, 18.43)	10.2 (8.25, 18.6)	13.12 (9.02, 18.78)	10.24 (7.12, 15.23)	.528
K (mmol/L)	4.14 (3.79, 4.5)	4.13 (3.75, 4.47)	4.02 (3.75, 4.58)	4.18 (3.8, 4.46)	4.19 (3.81, 4.45)	4.1 (3.9, 4.56)	.981
Na (mmol/L)	139.8 (137.8, 141.5)	139.2 (137.95, 142.6)	139.8 (138.4, 141)	139.25 (137.8, 141.03)	140.1 (137.9, 141.87)	139.69 (136.38, 142.45)	.747
BUN (mmol/L)	3.95 (3.15, 4.94)	3.82 (3.15, 4.33)	3.7 (2.86, 4.4)	3.7 (3.13, 4.94)	4.34 (3.28, 5.45)	5.12 (3.6, 7.35)	.000**
Cr (µmol)	67.5 (56, 80)	70.5 (56.1, 81.75)	67 (57.1, 81.5)	69.15 (55.08, 79.38)	64.2 (55.75, 76.7)	71.45 (56.75, 81.8)	.562
LDH (U/L)	177.5 (153, 219.25)	169 (147, 209.5)	175 (146.5, 203.75)	187 (160.75, 228.25)	172.5 (148.5, 196.5)	198 (159, 303)	.07
Mb (µg/L)	36 (30, 57)	37.35 (30, 43.73)	35 (30, 47.9)	33.5 (30, 52.89)	39 (30, 61.63)	86.25 (41.15, 202.95)	.002**
Glu (mmol/L)	5.45 (4.95, 6.45)	5.13 (4.9, 5.52)	5.44 (4.75, 6)	5.62 (5.08, 6.94)	5.51 (5.08, 6.83)	5.43 (5.12, 9.31)	.229
ESR (mm/h)	20.45 (8, 44.88)	14.5 (6, 39.25)	19.3 (6, 40)	17 (11.25, 39.75)	20 (8, 44.13)	69.8 (30, 85)	.000**
CRP (mg/L)	5.19 (5, 16.6)	5 (5, 13.62)	5 (2.51, 11.74)	5 (3.69, 18.6)	5.6 (5, 16.61)	13.9 (5, 48.55)	.003**
PCT (ng/ml)	0.06 (0.05, 0.1)	0.05 (0.03, 0.1)	0.06 (0.05, 0.1)	0.05 (0.05, 0.1)	0.05 (0.05, 0.1)	0.1 (0.07, 0.13)	.000**

*Note*: *p < .05, **p < .01.

Abbreviations: ALT, alanine aminotransferase; APTT, activated partial thromboplastin time; AST, aspartate aminotransferase; BUN, urea nitrogen; CK, creatine kinase; CK‐MB, creatine kinase isoenzyme; Cre, creatinine; CRP, C‐reactive protein; DD, D‐Dimer; ESR, blood cell sedimentation rate; Glu, random blood glucose; Hb, hemoglobin; L, lymphocyte; LDH, lactate dehydrogenase; Mb, myoglobin; N, neutrophil; PCT, procalcitonin; Pt, platelets; PT, Prothrombin time; TBIL, Total bilirubin; WBC, white blood cell.

**Figure 3:**
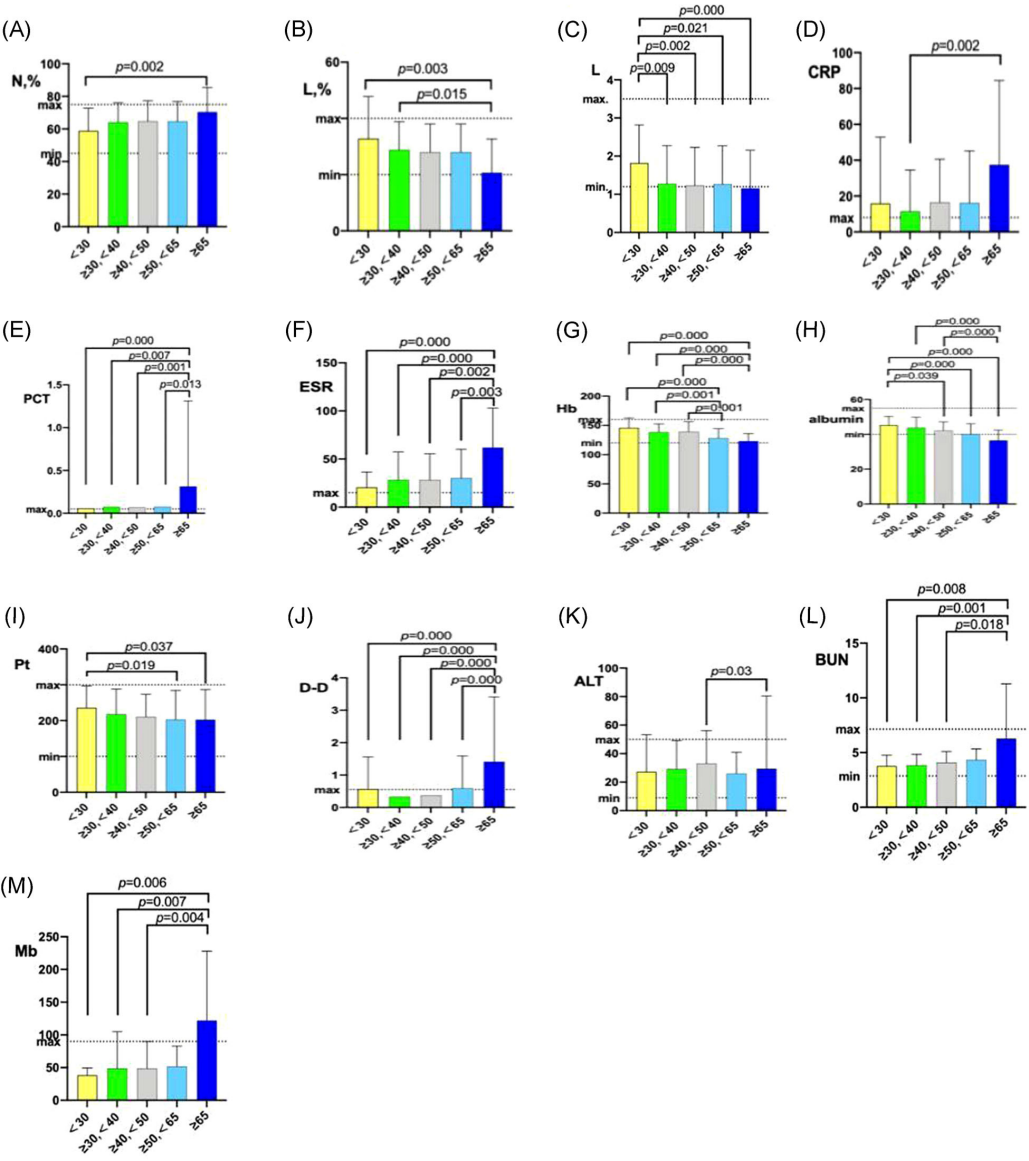
Comparison of Lab tests according to age groups

Measurements such as BUN (5.12 vs. 3.82, *p* = .008; 5.12 vs. 3.7, *p* = .001; 5.12 vs. 3.7, *p* = .018) and Mb (86.25 vs. 37.35, *p* = .006; 86.25 vs. 35, *p* = .007; 86.25 vs. 33.5, *p* = .004) of age ≥65 were significantly higher than those of age <30, 30–40 and 40–50. In addition, levels of DD of age ≥65 was significantly higher than those of age <30, 30–40 and 40–50 and 50–65 (0.66 vs. 0.29, *p* < .001; 0.66 vs. 0.28, *p* < .001; 0.66 vs. 0.31, *p* < .001; 0.66 vs. 0.35, *p* < .001). Levels of platelets of age ≥65 and 50–65 was significantly lower than those of age <30 (175.5 vs. 245, *p* = .037; 184 vs. 245, *p* = .019) (Table 2 and Figure 3). These changes suggesting potentially heart, kidney and coagulation function damage in the oldest age group. The levels of WBC, N, PT time, APTT, AST, CK, CKMB, TBIL, K, Na, Cr, LDH, and Glu of the groups were not statistically different among different age groups.

### 3.4 Medical treatments and outcomes of COVID‐19 among the age groups

80% received Interferon alpha inhalation (239/299), 76% of the patients received Lopinavir/ritonavir (228/299), 57% received Arbidol (169/299), and 52% received Lianhua Qingwen Capsule treatment (Chinese medicine). Combination use of Arbidol, Lopinavir/ritonavir or Interferon alpha inhalation was also common. Empirical antibiotic treatment was used when bacterial infection was suspected, which may reference for elevated Neu, PCT value and sputum. 42% were received antibiotic (126/299). Percentage of corticosteroid (17/57, 43%) and Immunoglobulin (19/56, 48%) treatment was highest in age ≥65 (Table 3).

**Table 3:** Medical treatments and outcomes of COVID‐19 among the age groups

	Total	<30	≥30,<40	≥40,<50	≥50,<65	≥65	
	*n* = 299	*n* = 41	*n* = 75	*n* = 74	*n* = 69	*n* = 40	*p* value
Onset treatment	4 (1, 6)	3 (1, 5)	3 (1, 7)	4 (1.75, 7)	4 (2, 6.75)	3 (1, 6.5)	.687
Arbidol+lopinavir/ritonavir+interferon alpha inhalation	120 (40%)	13 (32%)	28 (37%)	29 (39%)	32 (46%)	18 (45%)	.558
Arbidol+Lopinavir/ritonavir	134 (45%)	15 (37%)	30 (40%)	34 (46%)	36 (52%)	19 (48%)	.479
Arbidol+interferon alpha inhalation	141 (47%)	16 (39%)	32 (43%)	32 (43%)	40 (58%)	21 (53%)	.211
Lopinavir/ritonavir+interferon alpha inhalation	189 (63%)	24 (59%)	47 (63%)	47 (64%)	45 (65%)	26 (65%)	.966
Arbidol	169 (57%)	19 (46%)	37 (49%)	42 (57%)	46 (67%)	25 (63%)	.148
Lopinavir ritonavir	228 (76%)	30 (73%)	56 (75%)	60 (81%)	54 (78%)	28 (70%)	.683
Interferon alpha inhalation	239 (80%)	34 (83%)	58 (77%)	55 (74%)	58 (84%)	34 (85%)	.498
Lianhua Qingwen capsule	155 (52%)	21 (51%)	38 (51%)	39 (53%)	29 (42%)	28 (70%)	.091
Antibiotics	126 (42%)	13 (32%)	28 (37%)	30 (41%)	31 (45%)	24 (60%)	.089
Corticosteroid	57 (19%)	3 (7%)	11 (15%)	12 (16%)	14 (20%)	17 (43%)	.001
Immunoglobulin	56 (19%)	2 (5%)	6 (8%)	15 (20%)	14 (20%)	19 (48%)	.000**
Severity (1)							.000**
Mild	32 (10.7%)	5 (12.2%)	11 (14.67%)	10 (13.51%)	5 (7.25%)	1 (2.5%)	
Moderate	231 (77.26%)	35 (85.37%)	60 (80%)	57 (77.03%)	54 (78.26%)	25 (62.5%)	
Severe	26 (8.7%)	1 (2.44%)	3 (4%)	4 (5.41%)	10 (14.49%)	8 (20%)	
Critical	10 (3.34%)	0 (0%)	1 (1.33%)	3 (4.05%)	0 (0%)	6 (15%)	
Severity(2)							
Mild or moderate	263 (87.96%)	40 (97.56%)	71 (94.67%)	67 (90.54%)	59 (85.51%)	26 (65%)	.000**
Severe or critical	36 (12.04%)	1 (2.44%)	4 (5.33%)	7 (9.46%)	10 (14.49%)	14 (35%)	
ICU	15 (5%)	0 (0%)	3 (4%)	2 (3%)	3 (4%)	7 (18%)	.003**
ARDS	26 (9%)	0 (0%)	3 (4%)	5 (7%)	6 (9%)	12 (30%)	.000**
SHOCK	6 (2%)	0 (0%)	1 (1%)	1 (1%)	0 (0%)	4 (10%)	.006**
Hospitalization days	17 (12, 23.5)	16 (11.25, 20.75)	15 (11, 21)	16 (10, 22.25)	18 (13, 25.5)	17 (12,24)	.189
Treatment negative	13 (9, 19)	11.5 (8,18)	10 (7,18)	11 (8,18.5)	16 (10,22)	15.5 (10, 21)	.009**
Outcome							
Cured and discharged	285 (95.32%)	41 (100%)	74 (98.67%)	72 (97.3%)	67 (97.1%)	31 (77.5%)	.000**
On medical treatment in hospital	12 (4.01%)	0 (0%)	1 (1.33%)	2 (2.7%)	2 (2.9%)	7 (17.5%)	
In‐hospital death events	2 (0.67%)	0 (0%)	0 (0%)	0 (0%)	0 (0%)	2 (5%)	

*Note*: *p < .05, **p < .01.

Abbreviations: ARDS, respiratory distress syndromes; ICU, intensive care unit.

In particular, mild type accounted for 10.7% (32/299), moderate type accounted for 77.26% (75/299), severe type accounted for 8.7% (10/299), critical type accounted for 3.34% (4/299). Proportion of severe or critical type was 2.44%, 5.33%, 9.46%, 14.49%, and 35% in patients with age <30, 30–40, 40–50, 50–65, ≥65, respectively (*p* < .001). The medium time from onset treatment to throat swab turn negative was 15.5 days in age ≥65, 16 days in age 50–65, and 11 days in age <30, 10 days in 30–40, 11.5 days in 40–50 (Table 3).

At this point, ICU admission rate was 0%, 4%, 3%, 4% and 18% in age <30, 30–40, 40–50, 50–65, ≥65 (*p* = .003). ARDS (respiratory distress syndromes) rate was 0%, 4%, 7%, 9% and 30% in age <30, 30–40, 40–50, 50–65, ≥65 (*p* < .001). Rate of shock was 0%, 1%, 1%, 0% and 10% in age <30, 30–40, 40–50, 50–65, ≥65 (*p* = .006). 285 patients (95.32%) were cured and discharged, 12 patients (4.01%) was still on medical treatment in hospital, 2 patients (0.67%) died because of respiratory failure (*p* < .001). On medical treatment rate was 0%,1.33%, 2.7%,2.9% and 17.5% in age <30, 30–40, 40–50, 50–65, ≥65. There were no difference in time from symptom onset to initial treatment and hospitalization days among age groups (Table 3).

### 3.5 Comparison of clinical characteristics of COVID‐19 between mild/moderate type and severe/critical type

**Figure 4:**
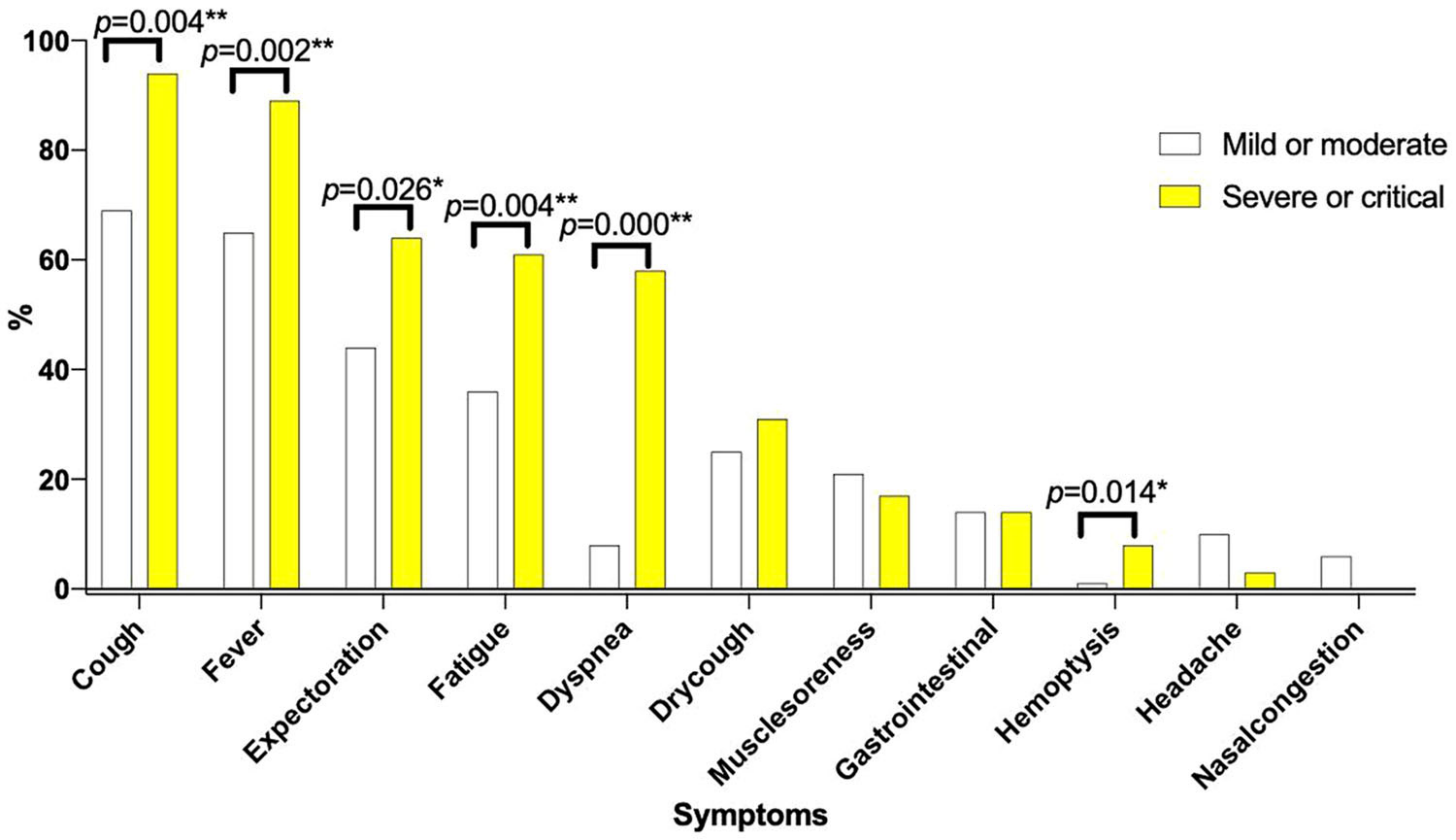
Symptoms of COVID‐19 according to mild/moderate and severe/critical type

Patients developed to severe or critical type were older than patients with mild or moderate type (57.72 vs. 44.06 *p* = .002). Comparison in Table 4 and Figure 4 were performed to determine factors associated with severe or critical type in overall COVID‐19 patients. Patients developed to severe or critical type have higher percentage of cough (94.44% vs. 69.2%, *p* = .004), fever (88.89% vs. 64.64%, *p* = .002), expectoration (63.89% vs. 44.11%, *p* = .026), fatigue (61.11% vs. 36.12%, *p* = .004), dyspnea (58.33% vs. 8.37%, *p* < .001), and hemoptysis (8.33% vs. 0.76%, *p* = .014) symptom than patients with mild or moderate type (Figure 4).

**Table 4:** Comparison of clinical characteristics of COVID‐19 between mild or moderate type and severe or critical type

	Mild or moderate	Severe or critical	
	*n* = 263	*n* = 36	*p* value
Age	44.06 ± 14.81	57.72 ± 15.5	.000**
Sex	135 (51.33%)	23 (63.89%)	.157
Weight	50.25 ± 29.43	107.57 ± 221.5	.13
Height	138.14 ± 50.22	114.33 ± 55.11	.018*
BMI	23.77 ± 3.8	24.02 ± 3.03	.708
Total number of chronic disease	0 (0, 1)	1 (1, 2)	.000**
Temp	36.7 (36.5, 37.1)	36.9 (36.5, 37.65)	.133
HR	86.38 ± 11.28	97.41 ± 10.34	.000**
RR	20 (19, 20)	20 (20, 22)	.000**
Chronic disease	77 (29.28%)	28 (77.78%)	.000**
DM	24 (9.13%)	11 (30.56%)	.001**
hypertension	37 (14.07%)	16 (44.44%)	.000**
Chronic heart disease	6 (2.28%)	8 (22.22%)	.000**
Chronic pulmonary disease	11 (4.18%)	6 (16.67%)	.009**
Cancer	4 (1.52%)	1 (2.78%)	1
Chronic nervous system disease	6 (2.28%)	2 (5.56%)	.248
Chronic liver disease	5 (1.9%)	3 (8.33%)	.059
Chronic kidney disease	0 (0%)	1 (2.78%)	.12
Endocrine and immune disease	4 (1.52%)	1 (2.78%)	1
Bilateral involvement on chest radiographs	204 (77.57%)	32 (88.89%)	.118
Epidemiological history			
Primary case	107 (40.68%)	10 (27.78%)	.008**
Secondary case	101 (38.40%)	10 (27.78%)	
Unknown case	55 (20.91%)	16 (44.44%)	
Hospitalization days	17.18 ± 7.83	20.89 ± 9.28	.011*
Treatment Negative	14.23 ± 8.27	16.29 ± 6.83	.161
Onset treatment	4.68 ± 4.37	5.67 ± 4.74	.209
ICU	6 (2.28%)	9 (25%)	.000**
ARDS	7 (2.66%)	19 (52.78%)	.000**
SHOCK	1 (0.38%)	5 (13.89%)	.000**
Outcome			.000**
Cured and discharged	258 (98.10%)	27 (75%)	.000**
On medical treatment in hospital	5 (1.9%)	7 (19.44%)	
In‐hospital death events	0 (0%)	2 (5.56%)	
Arbidol	141 (53.61%)	28 (77.78%)	.006**
Lopinavir ritonavir	197 (74.9%)	31 (86.11%)	.138
Interferon alpha inhalation	207 (78.71%)	32 (88.89%)	.153
Lianhua Qingwen Capsule	130 (49.43%)	25 (69.44%)	.024*
Antibiotics	95 (36.12%)	31 (86.11%)	.000**
Corticosteroid	32 (12.17%)	25 (69.44%)	.000**
Immunoglobulin	29 (11.03%)	27 (75%)	.000**

*Note*: *p < .05, **p < .01.

Abbreviations: ARDS, respiratory distress syndromes; BMI, body mass index; DM, diabetes mellitus; HR, heart rate; ICU, intensive care unit; RR: respiratory rate; Temp, temperature.

In general, patients developed to severe or critical type have higher percentage with at least one chronic disease (77.78% vs. 29.28%, *p* < .001), DM (30.56% vs. 9.13%, *p* = .001), chronic heart disease (22.22% vs. 2.28%, *p* < .001) and chronic pulmonary disease (16.67% vs. 4.18%, *p* = .009) compared to patients with mild or moderate type. Primary and secondary case accounts for 40.68% and 38.40% in patients with mild or moderate type, however, this proportion was 27.78% and 27.78% in severe or critical type. Noticing that 44.44% of severe or critical type was infected with unknown origin of infected.

Besides, relatively high percent of drugs was prescribed in severe or critical type than mild or moderate type. Obviously, severe or critical type have longer hospitalization days (20.89 vs.17.18, *p* = .011), higher rate of ICU admission (25% vs. 2.28%, *p* < .001), ARDS (52.78% vs. 2.66%, *p* < .001), and shock (13.89% vs. 0.38%, *p* < .001) than mild or moderate type. Still on medical treatment rate was 19.44% in severe or critical type and 3.07% in mild or moderate type. In‐hospital death cases were two in severe or critical type and none in mild or moderate type (Table 4).

### 3.6 Factors for severe or critical type in COVID‐19 patients

In a binary logistic regression analysis, we found old age (OR: 1.055, 95% CI: 1.016–1.095, *p* = .006), heart rate (HR) in admission (OR: 1.085, 95% CI: 1.030–1.144, *p* = .002), respiratory rate (RR) in admission (OR: 1.635, 95% CI: 1.093–2.431, *p* = .017), and history of chronic heart disease (OR: 56.038, 95% CI: 2.764–1136.053, *p* = .009) showed independent associations with severe or critical type (Table 5).

**Table 5:** Binary logistic regression analysis of factors for severe or critical type in COVID‐19

	*β*	*SE*	Wald	Exp(β)	95% CI	*p* value
Age	.053	0.019	7.695	1.055	1.016–1.095	.006**
HR	.082	0.027	9.416	1.085	1.030–1.144	.002**
RR	.488	0.204	5.733	1.635	1.093–2.431	.017*
history of chronic heart disease	4.026	1.535	6.876	56.038	2.764–1136.053	.009**

*Note*: Age, HR in admission, RR in admission, history of DM, hypertension, chronic heart disease, chronic lung disease, epidemiological history were included as covariates. *p < .05, **p < .01.

Abbreviations: CI, confidence interval; HR, heart rate; RR: respiratory rate.

### 3.7 Discussion

To the best of our knowledge, our study is the first to show that high heart rate in admission, high respiratory rate in admission are related to the progression of the severe or critical type of COVID‐19. And we also found that patients with a history of chronic heart disease had a more than a 56 times higher risk for severe or critical type of COVID‐19 than those without a history of chronic heart disease.

In this study, the fatality of COVID‐19 infection was 0.67%. The outcome and clinical characteristics of patients outside of Wuhan differed from those initially reported in patients in Wuhan.^11^, ^12^ Meanwhile, in our study, severe or critical type accounted for 12.04% (36/299), and most of the patients were mild or moderate (263/299 = 87.96%). The trends are relatively consistent across the major reporting states in China.^12^ Until March 23, 2020, cumulative reported confirmed cases was 1018 in Hunan province which had no confirmed cases or no new confirmed cases for 14 consecutive days and was listed as low‐risk urban areas in China. From March 23, 2020 to July 17, 2021, 50 newly primary patients with COVID‐19 was came from abroad in Hunan province. There are currently 1068 patients with COVID‐19 in Hunan Province.^3^ This reflects effective treatment and public health response.

Aging is associated with a progressively weakened immune system, decreased lung performance, and a bad outcome in CAP. Although we can not assess the susceptibility of COVID‐19 and old age, but we found old age was significantly associated with severe or critical type. In recent published studies, the patients in the progression group were significantly older than those in the disease improvement/stabilization group.^13^ What' s more, for critically ill adult patients infected with COVID‐19 who were admitted to the ICU in Wuhan, researchers found older patients (>65 years) with comorbidities and ARDS are at increased risk of death.^14^ These results indicate that age‐related risk factors for a bad outcome should be considered when developing effective medical treatment strategies for older patients.

The patients with severe or critical type also more frequently had cough, fever, expectoration, fatigue, dyspnea, and hemoptysis symptoms. Physicians need to be alert to these symptoms. COVID‐19 infection in elderly seems to be a little different with elderly CAP, as the elderly with bacterial pneumonia may lack the typical acute respiratory symptoms, but with gastrointestinal symptoms.^6^, ^15^ In our study, for elderly, dyspnea symptoms were more common in elderly and respiratory symptom like cough, dry cough, expectoration, fatigue were common symptoms in elderly. Despite this, altered mental status, a sudden decline in functional capacity, and worsening of underlying diseases is still important for differentiate severe cases in COVID‐19.

Comorbidity is another important risk factor for pneumonia.^16^ In our study, patients who developed severe or critical type were more likely to have underlying medical comorbidities, including DM, hypertension, chronic heart disease and chronic pulmonary disease, compared to mild or moderate type. In the logistic analysis, history of chronic heart disease were independently associated with severe or critical type in the regression analysis. There were also an increase trend in medical comorbidities (DM, hypertension, chronic heart and pulmonary disease) with advancing age groups. We also noticed a twofold increase of Mb levels in elderly group compared to other age groups. Coexisting chronic diseases are likely to work in a synergistic manner, affecting the overall health and adaptability of patients. As demonstrated by other studies that myocardial injury is significantly associated with fatal outcome of COVID‐19.^17^, ^18^

Physicians and patients probably need to be alert to high or increased heart rate, respiratory rate with COVID‐19. As HR and RR were all easily available data before patients visit physicians, clear awareness of these factors and understanding of their predictive value would help to predict the disease severity, which may further customize the medical care being provided when needed. The initial vital signs and the results of basic laboratory examinations and chest imaging are critical information that is required for clinicians to rapidly judge patients' health condition, in particular the severity of acute disease. Although there were significant increase in levels of N%, DD, ALT, BUN, Mb, ESR, CRP, and PCT and significant decrease in levels of L%, L, Pt, Hb, and albumin in old age group, the majority of these values were still in the normal limit of reference, suggesting subtle and subclinical changes of many blood lab tests occur with severe or critical type of infection. The association between old age and severe or critical type COVID‐19 might be due to reduced immune function, as reflected by increased neutrophil and decreased lymphoncyte percentages, and poor nutrition, reflected by decreased Hb, albumin; inflammatory factor storm, reflected by increased ESR, CRP, and PCT; impaired coagulation function, liver, kidney, heart function, reflected by increased DD, ALT, BUN, Mb and decreased Pt. Recent study also demonstrated decreased albumin is a risk factor for the progression of COVID‐19 pneumonia.^13^ And anticoagulant therapy appears to be associated with better prognosis in severe COVID‐19 patients meeting sepsis‐induced coagulopathy or with markedly elevated D‐dimer.^19^ The author thinks immune regulation, nutrition improvement, inflammatory response control may be helpful for treatment.

As novel coronavirus were newly spread virus, there are many clinical uncertainty. Choosing an effective antivirus as well as timely treatment during the management of COVID‐19 may may improve outcomes, including direct medical, social, and economic. Randomized controlled double‐blind clinical trials may provide treatment insights for COVID‐19.

The limitation of our study was the limited cases. National wide study with different races will be more more representative and validate the findings of the present study. Second, although we checked vital signs, epidemiological history, chronic disease histories, we only find old age (OR: 1.055, 95% CI: 1.016–1.095, *p* = .006), heart rate in admission (OR: 1.085, 95% CI: 1.03–1.144, *p* = .002), respiratory rate in admission (OR: 1.635, 95% CI: 1.093–2.431, *p* = .017) and history of chronic heart disease (OR: 56.038, 95% CI: 2.764–1136.053, *p* = .009) were risks for more severe disease. However, beyond these factors listed above, previous study have reported several other factors, like viral exposure virulence, such as viral subtype and virulence, viral load that may influence disease severity.^20^ In the future, we may need to expand epidemiological data to further verify whether these possible related factors are related to more serious diseases.

## 4 CONCLUSION

Patients with a history of chronic heart disease had a more than a 56 times higher risk for severe or critical type of COVID‐19 than those without a history of chronic heart disease (OR: 56.038, 95% CI: 2.764–1136.053, *p* = .009). Age, heart rate in admission, respiratory rate in admission are related to the progression of the severe or critical type of COVID‐19 and thus provide prognostic information and help clinicians in alerting high‐risk patients.

## CONFLICT OF INTERESTS

The authors declare that there are no conflict of interests.

## AUTHOR CONTRIBUTIONS

**Wei Cheng and Yating Peng**: generated the hypothesis, directed the implementation. **Yating Peng, Wei Cheng, and Aiyuan Zhou**: contribute to statistical analyses and wrote the manuscript. **Ling Lin, Xucai Liao, Dingding Deng, Peng Huang, Wenlong Liu, Mingyan Jiang, Xudong Xiang, Qingcui Shuang, Shan Cai, Ping Chen, Xucai Liao**: supervised the field activities and edited the manuscript. All authors read and approved the final manuscript.

## Data Availability

The data that support the findings of this study are available upon reasonable request from the corresponding author Ping Chen.
